# The Significance of Albumin in the Urine

**Published:** 1914-06

**Authors:** 


### BUFFALO MEDICAL JOURNAL

Volume 69 JUNE, 1914 No. 11

### ORIGINAL ARTICLES

### The Significance of Albumin in the Urine.

A Symposium Conducted by Section IV of the Rochester Academy of Medicine on Wednesday evening, April 8, 1914. The Chairman of the Section, Dr. John M. Swan, in the chair.

**Contents**

1. The Chemical Tests for Albumin.
   Dr. Charles R. Witherspoon.
2. The Significance of Albuminuria in the Acute Infectious Diseases. Dr. John R. Williams.
3. The Significance of Albuminuria in Chronic Diseases:
   (a) Of the Respiratory System.
   Dr. E. G. Whipple.
   (b) Of the Circulatory System.
   Dr. N. W. Soble.
   (c) Of the Genito-Urinary System.
   Dr. W. F. Plumley.
   (d) Of the Alimentary System.
   Dr. W. V. Ewers.
   (e) Of the Nervous System.
   Dr. E. L. Hanes.
4. The Significance of Albuminuria to the Surgeon.
   Dr. C. W. Hennington.
5. The Significance of Albuminuria to the Obstetrician.
   Dr. W. M. Brown.
6. The Significance of Albuminuria to the Ophthalmologist.
   Dr. A. C. Snell.

### The Chemical Tests for Albumin.

Charles R. Witherspoon, M. D.

Serum albumin alone is of clinical importance; the Bence Jones Proteid occurs rarely but is distinguished by the routine application of tests for serum albumin; the detection and isolation of other proteids is valueless,

The tests for albumin are two: The nitric acid test (Heller's) and the heat test. The more delicate tests are too delicate.

Routine examination requires the use of either of these tests with the application of the other in case albumin is found with the first. The technique of these tests is as follows: "The nitric acid test is best performed in a small wine glass. After filling this half full of urine, insert a small glass funnel to the bottom of the urine and gently pour in concentrated nitric acid. If albumin is present, a white ring forms at the junction of the acid with the urine, either immediately or in the course of ten minutes. If carefully performed this test is delicate enough for all clinical purposes, but since some of the albumoses give a similar precipitate, the boiling test should be used as a control whenever a positive reaction is obtained with nitric acid. None of the other rings, observable above or below, but not at the junction of the acid with the urine, is of any clinical importance.

The boiling test: To half a test tube full of urine add three or four drops of dilute acetic acid ,and boil the upper three-quarter inch of the urine. If albumin is present a white cloud appears. If the Bence Jones body is present a white cloud appears on heating, disappears on boiling, and reappears on cooling. In performing this test the addition of acetic acid as above described is absolutely necessary to prevent error." Cabot.

The urine should be at room temperature, the light should be daylight, and at least ten minutes should elapse before the final reading.

---

### The Significance of Albuminuria in Acute Infectious Diseases.

John R. Williams, M. D.

The significance of albumin in acute infections is strikingly illustrated in the following brief case report:

American, male, mechanic, 22 years of age, complaining of vomiting, headache, insomnia, and frequent urination. Patient apparently made good recoveries from measles, whooping cough, mumps, in addition to several attacks of tonsilitis—all of which he had before the age of nine. At ten years of age, patient was very sick with scarlet fever, at which time an albuminuria was noted. At the age of fourteen there is a history of severe tonsillar infection complicated by inflamed joints, middle ear diseases and edema of the limbs. Since then patient's history is that of one suffering with impaired kidneys. An examination of two days ago reveals a systolic blood pressure of 219, a diastolic pressure of 105, and a pulse rate of 105. An examination of 24 hour urine is as follows:

Amount 2640 cc. Sp. Gr. 1005.
Total solids 30 grams.
Moderate amount of albumin.
No sugar; no diacetic acid.
A few blood cells; a large number of hyalin and granular casts.

A functional kidney test showed at the end of two hours an elimination of 26% of the phenol-sulphone-phthalein.

This case is an extreme illustration of the damage done to the kidneys by the repeated assaults of the acute infections of childhood.

Within the past few months thirty individuals suffering from renal insufficiency have come under my observation. Sixteen of these patients were over fifty years of age; nine of them were over thirty-five years. The phthalein functional kidney test showed an impairment of from twenty to sixty per cent.* In twenty, abnormally high systolic blood pressure was found. Eighteen of them exhibited clearly defined evidence of renal destruction by the presence of albumin and casts in the urine. In fifteen of these thirty cases the most significant etiological factor is the history of one or more acute infections, in which tonsilitis, scarlet fever, measles, and whooping cough preponderate.

These cases suggest that the acute infections of childhood frequently become the renal insufficiencies of adult life. Osler states that the kidneys are damaged in from ten to twenty per cent. of the acute exanthemeta of childhood. No one can say in what proportion this damage is permanent, but we have reason to believe that when the parenchyma of the kidneys is once destroyed, it is never again replaced. Accordingly, it should be the duty of the physician to watch vigilantly for evidence of renal diseases in the acute infectious diseases. This evidence may appear in a striking form as an acute hemorrhagic nephritis in which the small amount of urine passed will be heavily laden with blood, albumin, and casts. Or in a less serious type of case in which there may be a very small amount of albumin, but in which an edema and other evidence of renal insufficiency will be evident. A third and still more difficult type of case is the one in which little or no albumin

*Phenol-sulphone-Phthalein injected intravenously. Kastle-Roberts colorimeter used.

is to be detected and in which there may be at times complete absence of evidence of renal diseases. In this third type of case it may only be detected after weeks or months of careful observation. The symptoms of anemia, languor, and other illy defined ailments finally suggest to the physician that he is dealing with a case of renal impairment.

In conclusion, while the presence of albumin in the urine of a patient with an acute infectious disease may be a transient phenomenon, in many cases it is indication of renal destruction, which may ultimately prove to have a serious prognostic significance. Accordingly, the urine of every patient ill with an infectious disease should be carefully watched for evidence of kidney disease and the patient should be safeguarded by proper dietary treatment, rest, medical treatment, to limit renal destruction.

---

### The Significance of Albuminuria in Chronic Diseases of the Respiratory System.

E. G. Whipple, M. D.

I regret very much that from the literature or from my personal experience, I can find very little of importance on this subject. In treating the subject I will assume the term albuminuria to mean the commonly accepted clinical application of the word which includes a large number of related substances not strictly albumin but which, alone or combined, are of clinical significance and may be included under the broad term albumin.

The most common and most important chronic respiratory disease is pulmonary tuberculosis and albuminuria plays a small role in any phase of the consideration of the disease.

Dorland's Dictionary, under the term albuminuria defines "Albuminuria pretuberculosis," as a condition preceding clinical signs of the disease. I personally, do not believe such a condition exists, in reference to pulmonary tuberculosis, as a clinical entity. There is no doubt but that the presence of albumen in the urine especially if interpreted as a definite nephritis, lowers the resistance of any individual and so renders him more susceptible to tuberculosis—or any other infectious disease, for that matter. Barringer quotes a series of 396 individuals examined for life insurance, in which he found albumen; some with albumen alone, others with hyaline casts, others with granular casts, some with both hyaline and granular casts in addition to the albumen. Of these 396 cases 25 died within ten years and only three died of neuphritis while

8 died of pulmonary T. B. He draws the conclusion that the presence of albumen with or without casts predisposes to T. B. So it does and so does any pathological condition which lowers the resistance of the individual.

Not a few articles have been written in reference to Orthostatic Albuminuria and its relation to Pul. T. B. There seems to be many conflicting opinions. Ludeke and Sturm examined the urine of 140 T. B. patients. Sixty were incipent, 50 in the second stage and thirty in the third stage, 103 of the 140 showed a positive re-action to albumen after being on their feet for one hour. There was no trace in 8 healthy controls. He concludes that orthostatic albuminuria in these cases was due to the action of toxins and an auto-intoxication in some few instances. His most important conclusion was that orthostatic albuminuria may reveal incipient T. B., when the albumen can not be explained in any other way. Gamolitsky states that orthostatic albuminuria does not occur except in functional disturbance of the kidneys, especially from the toxins from infectious diseases. Arnold, on the other hand, examined the urine of 44 patients with chronic T. B. of the skin—8 with psoriasis and 33 with syphilis and found no trace of albumin in the skin diseases but present in the early and untreated cases of syphilis as often as in T. B. He concludes that it is NOT to be regarded as a sign of T. B. but rather a sign of a chronic infection and intoxication and not of specific import. It would seem to me that the latter is the better conclusion and I do not consider orthostatic albuminuria of any importance in the diagnosis of Pul. T. B.

When forced feeding of T. B. patients was considered good treatment, many cases developed albumin in the urine with and without hyaline and granular casts and their general condition was no doubt made worse. Feeding an inactive patient on a large amount of proteid food and with large amounts of liquid often did harm but most of these cases which have come under my observation cleared up very readily under a proper dietary unless they were advanced cases. This form of albuminuria may be seen in any stage of Pul. T. B. and is of importance if continued over a period of two weeks. It is also of importance in that it shows that Pul. T. B. lessens the functional activity of all organs and so it has assisted us in our treatment—showing the fallacy of imposing an extra burden upon an already weakened organ.

Albumen is found in the urine of T. B. patients most often in the terminal stages of the disease when evidence of a mixed infection is apparent and the system is overwhelmed by the toxins of the disease. It is usually accompanied by either hyaline or granular casts or both and is of importance only in prognosis, usually meaning that death will soon follow. There is usually oedema of the ankles and face, although this oedema does occur in the terminal stages, due largely to the weakened circulation and when no evidence of a kidney lesion can be found.

The consideration of albuminuria in other chronic respiratory diseases is of less importance than in T. B. In bronchiectasis and Pul. abscess albumen occurs especially in the terminal stages when the system is poisoned by the absorption of toxins and it is again only a manifestation of an intoxication which may occur from practically any chronic infectious disease.

In asthma when we find albumen, especially with casts we should not consider the asthma as anything other than a local manifestation of a kidney condition. If it is the so-called bronchial asthma the urine shows nothing relating directly to the disease. In chronic bronchitis and chronic pleurisy the urine shows nothing of importance to us.

Albumen in chronic respiratory diseases, then, in almost every instance, is a manifestation of an intoxication from the infecting disease. Occasionally from overtaxing an already weakened organ albumin may be seen, as for instance when a resting patient is over-fed, especially on a diet of proteids with a large quantity of fluid.

This responds to treatment in early cases. Pul. T. B. is the most important chronic respiratory disease and albumen occurs most often in the terminal stage and is of grave significance. There is no conclusive evidence that orthostatic albuminuria is of value in the diagnosis of Pul. T. B.

---

### The Significance of Albuminuria in Chronic Diseases of the Circulatory System.

N. W. Soble, M. D.

Mr. President and Fellows of the Academy:—

In considering the significance of Albuminuria in Circulatory disease, we must first of all bear in mind the following facts which are now firmly established. In the first place we must remember that one of the most important functions of the normal kidney is its refusal of passage of proteids from the blood, whilst it allows free passage of the end products of metabolism. Whenever we find Albumen in the urine it signifies first of all that some of the blood proteids are not being held back as they should be. Whether the mere presence of a more or less marked trace of Albumen in the urine is an indication of disease in quite another question. Thomas Fuller wrote ''Reasons drawn from the urine are as brittle as the Urinal.'' Just as structural diseases of the kidney may occur without albumen, so may Albuminuria exist quite apart from any structural disease. Von Leube says that Albumen, even a trace, when persistent, especially in a man over forty, is usually due to beginning Endarteritis, affecting not only the general vascular system, but those of the kidney. On the other hand Osler says,—''Albuminuria, in the case of a man over forty with or without a few hyaline casts, is not of much significance except as an indication that his kidneys. like his hair, are beginning to turn gray with age. This question has been largely discussed by many writers, notably Senatour, Von Noordin, Osler, Diulefoy. Whether there is such a condition as physiological albuminuria without any lesion of the circulatory system is as yet not fully decided. But the fact that albumin may exist in the urine in fairly well-marked quantities, and for a long period of time without any disturbance of health, may be accepted as true. It is not the purpose of this paper to discuss the question of

Physiologic albuminuria,
Intermittent albuminuria,
Cyclical albuminuria,
Digestive albuminuria,
Neuropathic albuminuria, nor Orthostatic albuminuria, as these would lead us entirely away from the principal subject of this discussion. Nevertheless we must not forget to mention that, even in the presence of Circulatory disease, we are compelled to differentiate the source of the albumen when present. Any inflammatory condition of the Genito-Urinary tract, not dependent upon general vascular disturbances will give albumen in the urine. I may mention Renal calculus,

Pyelitis,
Ureteritis,
Cystitis,
Prostatitis,
Seminal vesiculitis,
Urethritis, and in women, Vaginitis and Lucorrhoea from any source. Spermatozoa in the urine will also give us a trace of albumen. In Splanchoptosis with moveable kidney we often get a trace of albumen. And I need not mention all the infectious diseases, which as you well know are often accompanied by Albuminuria. It is important that we keep constantly in mind the fact that the kidney is acutely compensatory in its relation with the entire circulatory system, that it bears the burden of the blood-tide flowing through it; whenever albuminuria is associated with circulatory disease it signifies that compensation has failed, and that passive congestion of the kidney has occurred, and that the secretory function of the kidney has been altered: whether this alteration is due to increased pressure alone, or whether it is due to alteration of the structure of the epithelium of the tubules we cannot say. The possibility of these changes is enhanced by the variation in the blood pressure between the Renal artery arising from the abdominal Aorta on the one side, and the Renal vein discharging into the Vena Cava on the other. Thus any disturbance of the general circulation that is sufficient to alter the normal blood supply of the kidney will eventually interfere with the function of the kidney. Taking these facts into consideration, coupled with that we know of the hystology and physiological anatomy of the kidney, studies of which have recently been made by Landengren, it can be positively affirmed that other conditions remaining the same, the secretory function of the kidney will vary with the amount of blood passing through it. Recent experiments made by the above-mentioned author, prove that the kidneys are very vascular organs, that the quantity of blood which passes through them is very great. It is estimated that in one minute of time an amount of blood flows through them equalling the weight of the organ.

It is especially to be remembered at this point, that the flow of blood through the kidney, and therefore the secretory activity, is affected by conditions affecting the general arterial pressure; any change which will increase this difference in pressure between the blood in the renal Artery and the renal Vein will tend to augment the flow of blood through the glomerulus, and thus directly influence the secretory function. Hence it appears perfectly reasonable to expect, in a given condition of circulatory disturbance we must soon find manifestations in the urine by the presence of albumen in varying degrees. When one considers the rapidity of tissue change, the large mass of material which must be eliminated by the kidney in circulatory diseases, also the marked variation of blood supply, it is not strange that not only the function but the structure of the kidney should soon manifest alteration. With the advent of the new era in clinical medicine, and with the adoption of systematic routine examination of the urine in all cases, the profession is awakening to the fact that the kidney is often the first to give us warning of the existence of circulatory disturbance. While at all times, the mere presence of albumin alone in the urine can not be taken to mean the positive existence in quantities that are marked, and especially when accompanied by other elements of kidney structure must be taken by the clinician as an indication of the existence of circulatory disturbance. The value of this significance, or the exact amount of weight we are to attach to the finding will of course depend upon many other circumstances, some of which have been mentioned, but this much can be positively accepted, given a case of disease of circulation, with albumin in the urine, it becomes the duty of the clinical observer to proceed further in his search for additional evidence of structural kidney lesion.

The term ''Cardio-Vascular-Renal'' disease is so suggestive of the exact condition present in many instances, that it has come to be used as a distinct name for the disease of these three parts of the organism, the name itself being explanatory of the pathology and the prognosis.

In any given instance of Cardio-Vascular-Renal disease it is often doubtful for a long time whether the patient will finally succumb to Cardias disability, to Apoplexy from diseased arteries, to a general Arterio-sclerosis possibly of the Coronaries, or to Uraemia from Chronic Intestitial Nephritis. It is also many times impossible to tell where the disease first started. The beginning may be chronic Endarteritis with preceding hypertension, or a slowly developing chronic intestitial nephritis. In some instances an insufficient heart may cause so much passive congestion of the kidneys that they like the liver, may become the seat of secondary cirrhosis. But in all of these cases, the urinary examination, if made early and often, employing always a twenty-four hour specimen, will often give us albumin present in the varying quantities, and this will lead the careful clinician to extend his search further for other signs of structural kidney lesion. The Nephritic part of our triad is often very insidious in its origin, the first indication usually being prolonged hypertension, with its accompanying symptoms. For months there may be nothing abnormal in the urine and often the most careful search is necessary both for albumin and casts. It is wise to examine specimens passed early mornings and those passed immediately after meals. Quantitative tests of twenty-four hour specimens are valuable, and when albumin is found we must not rest here, we must satisfy ourselves that the albumin is not from some extraneous source.

### The Significance of Albuminuria in Chronic Diseases of the Genito-Urinary System.

W. F. Plumley, M .D.

I assume that the word albuminuria, as used in the subjects assigned this evening, refers to serum albumin and not to nucleoproteids and other albuminous substances sometimes found in urine.

We often hear the statement made that "albumin is accounted for by pus." This expression is almost invariably inaccurate and misleading. A large quantity of pus gives a reaction for only a trace of albumin. Pussy urines usually contain many bacteria and therefore it is difficult, and sometimes impossible, to filter them clear. If a turbid specimen gives a reaction it must contain more than a trace of albumin some of which is of kidney origin.

A profuse hemorrhage will give a reaction for albumin. The presence of casts in such a specimen justifies one in assuming that part of the albumin is from the kidney.

That albuminuria is a common accompaniment of chronic genito-urinary conditions, there is no doubt. That this albumin is in most cases of kidney origin, there also is little doubt. In the presence of chronic inflammation anywhere along the genito-urinary tract there is always the possibility of the infecting agent under favoring conditions working its way up to the kidney.

There seems to be little doubt that this does occur from time to time in some cases. Each small focus of inflammation in the kidney leaves a small scar. In time the kidney becomes a mass of scars and the same local and general changes and effects are found as in the case of ordinary red granular contracted kidney.

Any condition which causes a back pressure of the urine upon the kidney may be accompanied by albuminuria, e. g. Urethral stricture, enlarged prostate, pelvic tumors, floating kidney and obstruction to the ureters. Albumin of the above origin disappears when the obstruction is removed, except in prolonged cases, when the resulting kidney changes are much the same as in frequent kidney infections.

Irritation of the pelvis of the kidney may result in albuminuria. It is therefore a variable symptom in chronic tuberculosis of the pelvis, renal growths, renal calculus, etc.

There are some rare causes of albumin not necessarily included under my subject which I will merely mention: Systic disease of the kidney, new growths, thrombosis of the renal vein and inferior vena cava, infraction of the kidney.

In arriving at a conclusion as to the cause of albumin in any case the possibility of some genito-urinary factor should be considered. One is aided in making a diagnosis. First by obtaining the urinary history in detail, particularly regarding prolonged or repeated inflammatory conditions and urinary obstruction. Second by studying the sediment under the microscope. The presence of casts places the responsibility on the kidney. The kind of cast may give positive assistance particularly when it happens to be of the pus or blood variety. The character of the cellular elements in the sediment may help, but is apt to be misleading unless we remember that blood or pus must be present in large amount to account for more than a trace of albumin. In conclusion—A urine which shows more than a trace of albumin indicates the probability of one of the following conditions: nephritis—acute prostatitis or profuse hemmorhage. A trace may be found in (1) back pressure of urine on the kidney (2) a considerable quantity of pus (3) a moderate hemorrhage (4) subacute prostatitis (5) an irritation of the pelvis of the kidney.

---

### The Significance of Albuminuria in Chronic Diseases of the Alimentary System

W. V. Ewers, M. D.

Albuminuria is a fairly frequent complication of many digestive disturbances.

According to Gastoigne, there exists a real dyspeptic albuminuria. It is generally found in old dyspeptics, or those suffering with so-called nervous (?) dyspepsia, who drink from three to four leiters of milk a day. Gastoigne concludes that the albuminuria is directly due to the drinking of such large quantities of milk. He thinks he has proven this by the fact that he has found lac-albumin in the urine of these patients. He acknowledges, however, that the kidneys must be susceptible, as other dyspeptics upon the same diet do not secrete albumin. It seems to me that these are probably cases of digestive albuminuria, due to the taking, for these patients, of excessive quantities of proteins. This amount of milk represents from 99 to 132 grams of protein. A similar albuminuria is at times produced by the eating of large quantities of eggs, cheese or meat.

Chronic constipation is another condition frequently accompanied by albuminuria and cylinduria. Experiments which have been undertaken seem to show that the constipation produces a venious stasis, which, in turn, causes the albuminuria. The albumin will disappear after a thorough evacuation of the bowels. This would seem to confirm the correctness of the diagnosis in such conditions. In none of the reports of such cases are the blood pressure findings given, which, if stated, would help in removing any doubts one might entertain in regard to the correctness of the diagnosis.

Strumpell speaks of albuminuria accompanying a severe attack of gastric chrisis. We have at the General Hospital at the present time a case of gastric chrisis in which albuminuria and cylinduria are both present.

Gastric hemorrhage, and, according to Van Noorden, severe gastric cramps, are often accompanied by quite a marked albuminuria.

From 35% to 72% of all cases of carcinoma of the digestive tract show albuminuria.

Ewald speaks of the occurrence of albuminuria in acute disturbances of the digestive tract, especially acute intestinal occlusions.

After quoting the statements of others in regard to the digestive conditions in which albuminuria is observed and the quantities of albumin found, Boas makes the statement that according to his personal experience, it is the exception to find more than a trace of albumin in carcinoma of the oesophagus, stomach, the intestines or the liver.

My own rule has always been if I find albumen and casts, or albumin alone, in a mild case of digestive disturbance, to consider that the digestive disturbance is probably the complication and treat the case accordingly.

If we can be sure that the albuminuria is of digestive origin, the prognosis of course is very much better, for a digestive albuminuria per se, is of no significance. On account of the fact that nephritic patients are prone to digestive disturbances, we should scrutinize our cases very carefully before making a diagnosis of digestive albuminuria.

In severe gastric hemorrhage and in carcinoma, the origin of the albuminuria is probably the same as in these conditions arising in other parts of the body and needs no special comment here.

In conclusion, I think the safest rule to follow is before classifying an albuminuria as of digestive origin, to try and see if it will not fit into some of the other conditions under consideration this evening.

Remember, the only digestive disturbances commonly accompanied by albuminuria are chronic constipation, some cases of chronic dyspepsia, gastric hemorrhage, carcinoma,and intestinal obstructions.

### The Significance of Albuminuria in Chronic Diseases of the Nervous System

Edward L. Hanes, M. D., Rochester, N. Y.

Albuminuria in neurological practice is often a transitory symptom, without marked significance so far as the neurological process is concerned.

There are a number of brain and meningeal conditions wherein slight traces of albumin are occasionally observed, but in which the relationship of the underlying neurological state is often obscure, though at times it may be assigned to general altered conditions of the blood; while at others it appears to be part of a widespread condition throughout the body of rapid tissue metamorphosis.

It is a matter of occasional observation that episodes of emotional stress, or excitement, in otherwise apparently normal—or at most nervously organized—individuals often exhibit albuminuria during or following such upsets; while Strumpell refers to a condition of "cyclic" albuminuria in children who are sometimes healthy and strong, though more often nervous and anemic. These children often complain of headache, malaise, general weakness and languor, pain in the limbs, palpitation, anorexia, etc., and the albuminuria is usually to be traced to variations in bodily rest and activity—when quiet, no albumin, and the morning urine entirely negative, but after being out of bed for a short time and moving about actively, distinct albuminuria appears, especially after athletic exercises. In none of these cases is there suspicion of actual kidney lesion and in none are we able to postulate other significance to the appearance of albumin in these urines than constitutional predisposition to such reaction.

It may be said, therefore, that in general, albuminuric conditions are more or less transitory in primary nervous and mental disease, and have little special significance except as they point to individual idiosyncrasy or to accompanying constitutional involvement of the organism. There are some noteworthy exceptions, however, which interest us largely from the standpoint of differential diagnosis.

In the toxemias of almost every type wherein a selective or predominating reaction on nervous tissue occurs, albuminuria often serves to complicate the general nervous picture, just as in similar toxemic states manifesting their primary influence in other organs and portions of the body; thus in plumbism, and in mercuric poisoning, as well as in conditions of acute alcoholic intoxication, albumin is frequently noted among the urinary findings, and probably points to definite irritation of the secretory structures of the kidney. For similar reasons, also, in close analogy with the condition observed in the acute exanthematae, febrile meningetic states, particularly cerebro spinal meningitis, exhibit decided tendencies to albuminuria, often accompanied by casts.

As a result of the tremendous elaboration of nervous energy and accompanying tissue change noted in the spasmophilic and convulsive disorders, such as ex-ophthalmic goitre, tetany, delirium tremens without kidney lesion, in active maniacal outbursts of psychomotor excitement, and following epileptic convulsions, it is not at all unusual to observe albumin in the urine, serving merely as an index of the intensity of the metamorphic process.

In states of senile change, particularly those characterized by cardio-vascular sclerosis and high blood pressure, we have come to know that traces of albumin in the urine of these cases is a common finding, and I believe we usually regard it when present in small quantities as of no serious significance, except as it points to the kidneys as being involved in a rather widespread process. It is not surprising, therefore, in those extreme cases which still further emphasize the extensiveness of the process by inducing dementia of pathologic degree, to find in them evidence of albuminuria. In certain of the senile dementias, however, there appears to be a predeliction to sclerosis and atheroma of the cerebral vessels quite out of keeping with the evidence of vascular decay elsewhere throughout the system, and in these cases, frequently of almost complete annihilation of the faculties, one is astonished to find the urine practically normal on examination.

It is in these cases, too, of cerebral arterio-sclerosis with high blood pressure that we have come to expect the apoplexies, and here again in the pre-apoplectic stages when a number of neurotic symptoms present in the form of dizziness on rising or suddenly changing posture, headache, numbness of the extremities, mental haziness, somnolence or abnormal wakefulness, etc., the picture is frequently complicated by albuminuria; to be likewise observed at times on the occurrence of and following the stroke itself.

One of the most difficult tasks which we sometimes meet clinically is the differentiation between the coma and convulsions of uremia due to Bright's disease, and that occurring in the course of organic brain diseases in the nature of cerebral hemorrhage or embolism, pachymeningitis interna hemorrhagica, a fulminant attack of acute infectious meningitis, hemorrhage into the lateral ventricles, etc. I believe that, at times, such differentiation cannot be made antemortem, though we may commonly come to believe that the examination of the urine will reveal diagnostic characteristics. All these conditions are apt to exhibit albuminuria, though the presence of large quantities of the various casts may point, somewhat distinctly, toward the kidneys as the source of trouble. Undoubtedly Cabot's contention, that almost never do uremic symptoms of coma and convulsions appear out of a clear sky, holds much of value for us in approaching the problem of differential diagnosis, and we should remember that headaches, epileptiform attacks of mild intensity and transient duration, retinal symptoms, localized oedemas, tremors, nerve spasms and twitchings, states of altered mentality, etc., which we group as symptoms of chronic uremic poisoning, usually precede the appearance of actual coma and convulsions. I believe, nevertheless, when he states that he practically excludes uremia as a diagnostic possibility in conditions of coma in the absence of such preceding symptoms, he goes too far. In the great majority of these cases it is probable, however, that a careful history of onset, together with the urinary findings of albumin and casts will serve to clear up the diagnosis.

In certain conditions of obscure mental phenomena an examination of the urine with the determination of albuminuria may lead directly to a diagnosis, as in a recent puzzling case falling under my observation:

The patient, a powerfully built man, aged 55, and weighing about 220 pounds, was formerly an engineer, but gave up this occupation because of rheumatic difficulty. Two years ago he presented a well marked diabetic state, and underwent treatment for this disease until coming under my care, about October 1, 1913. At that time he was on a rigid carbo-hydrate free diet and his urine contained no sugar, nor other abnormal elements. He had recently developed prostrating headaches, with outbursts of emotional excitement against his wife, whom be accused of being interested in and on the watch for other men; these outbursts alternating with periods of sullen depression. He had partial insight into his condition but stated that when the spells came he could not seem to control himself. His blood pressure recorded 259 mm., and aside from this feature there was little to suggest the causation of his trouble. He was allowed a more liberal diet; his urine being tested for sugar from week to week with never even a trace being noted. Many tests for albumin were also made with negative results, till ultimately in January, of the present year, a single sample 24-hour urine gave distinct reactions by both the heat and nitric acid tests, while microscopic examination revealed considerable quantities of hyalin and granular casts. Since that occasion the urine has shown albumen reactions at rare intervals, though casts are nearly always present on examination.

In view of the cardiac hypertrophy, high blood pressure and urinary findings I have felt certain that we are dealing with a case of interstitual nephritis, complicated by mental symptoms, and the diagnostic significance of presence of albumin in the urine, even though negative tests were performed for weeks, is quite obvious.

---

### The Significance of Albuminuria to the Surgeon

C. W. Hennington, M. D.

It is now generally recognized that the mere absence of albumin, and of casts as well, by no means guarantees the kidneys to be in good condition. It, therefore, follows that in planning an operation, we must in certain cases keep the possible existence of a chronic nephritis in mind. We must look to other findings as low specific gravity, quantity, relative day and night amounts, and perhaps do a functional test as well. Above all we must look for clinical symptoms and signs of kidney disease especially as to evidence of hypertension with accompanying cardiac and vascular changes.

In fact, before the operations in which there is no necessity of haste, we should study the patient carefully, not alone as to the kidneys, but all the other organs as well, in order to determine the vital capacity or operative resistence. The recognition before operation of an impending acidosis and therefore by appropriate treatment preventing the distress and danger of this kind of post-operative vomiting, is also a matter worthy of our attention.

However, in this discussion we are concerned more particularly with the significance to be attached to the actual presence of an albuminuria. In other words, to what extent it increases the operative risks, both as to the ease and safety of immediate recovery and as to the effect on future health.

In acute surgical diseases there is often an accompanying albuminuria. But if the diagnosis is clear, operation should be done quite regardless of this albuminuria. It is probably merely secondary to the disease itself and need give no alarm, and certainly should not in any way delay the operation. A well chosen anaesthetic and energetic post-operative treat ment are all that is required.

But in all chronic surgical conditions which happen to have an associated albuminuria, we find ourselves challenged to investigate the condition in greatest detail. The most complete urine examinations are demanded in every case and perhaps a functional test of the renal capacity. We should find out how well the heart is able to cope with its load and perhaps attempt to discover the functional or reserve capacity of the heart muscle. The usual general investigation of the other organs is to be made. All this takes time but in the past we have erred by hurrying too much in these chronic cases to get them on the operating table. Thus, after every available fact is known, we can sum up that individual case and decide on the best course. It is seldom that we are compelled to choose on a clear cut question, "To operate or not." Usually there are several other alternatives such as a few days or weeks delay for medical treatment, or a change in the character of the proposed operation, or finally a consideration of the choice of various methods of anaesthesia, both general or local.

Failure to give sufficient attention to all these matters is sometimes followed by a death at operation or an early post-operative death. As a rule the results are not so extreme and self evident. In fact, they entirely escape the observation of the careless man who is not interested in these conditions. The most common results are post-operative distress, vomiting, restlessness or drowsiness, rapid pulse, pain in the back, and other more well known symptoms of kidney disturbance. At times we invite an acute anaesthesia nephritis with all its dangers both present and remote. Most often we are merely inconvenienced by a delayed convalescence and are not aware that our operation has lastingly impaired the kidneys and that patient's chances of a comfortable life. Matters such as these are well worth our consideration.

---

### The Significance of Albuminuria to the Obstetrician

W. M. Brown, M. D.

The answer is easy—in a way—It means danger. It is the "Stop, Look and Listen" on the Physiological Highway of Pregnancy and Labor. Modern social life and the changes in the progress of civilization have so altered and obstructed the channel of navigation in this condition that Nature has been forced to place various beacons for our guidance and the obligation is upon us to observe with an eternal vigilance and an understanding to interpret the signs that are so placed along the fairway, for on that vigilance and understanding will many times depend not only the life of mother and child but their future state of usefulness or dependence in the social cosmos.

But is the answer to our query easy? Having observed that there is albuminuria, what danger do we anticipate? From what does that danger arise? And further involved and implied in the question is what to do in the presence of the danger.

In order to answer these questions it is necessary to be able to differentiate the albuminuria in its etiological aspect. There are three chief forms of albuminuria in pregnancy. The most common and most important one is an albuminuria from the toxaemia of the pregnant condition; it is due to an acute change in the kidney caused by an effort to eliminate the toxin which causes eclampsia or the insufficiently oxydized proteins from the liver, the function of which has been interfered with by the toxin, or perhaps from the accumulated excreta from the foetus.

The albuminuria from chronic nephritis and cardiac change will probably antedate the pregnancy. The other form is due to the contamination by protein elements from the urinary tract below the kidney. It is agreed that in all cases, except those of the very rare form of functional or orthostatic albuminuria, the loss of albumin by the urine, unless it may be by contamination, means a compromise of the kidney function.

It has been said that the condition of pregnancy is the test of bodily fitness and that in this struggle the kidney is the organ of weakest resistance. So marked is this defect that von Leyden and the Germans have described what they call "The Kidney of Pregnancy."

In this condition the kidney is large, pale, soft, grayish yellow in color and microscopically shows fatty changes, but no infiltration of leukocytes or vascular changes. This change in the kidney takes place, in a greater or less degree, in a very large proportion of pregnancies, and as before stated it is due to a direct irritation from the toxin of pregnancy or its effects.

The first evidence of these changes may be the presence of small amounts of albumin in the urine and this, when found should be most carefully watched for the transition from the simple "Kidney of Pregnancy" to a grave nephritis may be sudden and violent and a kidney which allows the passage of albumin is some evidence of the virulence of the toxaemia and may be a guide as to our treatment.

Increasing amount of albumin, with the presence of tube or blood casts, together with a deficient nitrogen elimination, after a reasonable test of treatment, should indicate the necessity of emptying the uterus.

While the albuminuria from chronic nephritic change must be carefully watched and treated because it is apt to progress more rapidly in the pregnant condition, it has been observed that these patients are not more liable to have eclampsia than other women.

In them, beside the more rapid degeneration of the kidney and heart, the chief danger is one of placental changes, which interferes with the life and growth of the foetus and often results in an early miscarriage and death of the child.

The so-called functional albuminuria may be present before conception and is never aggravated by pregnancy.

True, albuminuria of pregnancy, as a rule, comes after the twenty-fifth week, and if of a considerable amount and increasing with casts should be vigorously treated, at the same time watching the associated signs of toxaemia. Retinal oedema with degeneration may result in defect or loss of vision if allowed to go one.

It is stated that albumin may be found in over thirty per cent. of pregnancies, if the finer tests are made, and fully fifty per cent. of women in labor will show a trace. This is due to renal anaemia from reflex vascular spasm.

Red infarcts of the placenta are generally associated with albuminuria, and, if well marked, cause imperfect development of the foetus, and sometimes cause its death. These infarcts are not, as a rule, found in eclamptic women, being noted only in those cases of true nephritic toxaemia.

Conclusions: Pregnancy with its toxaemia is a severe strain on the kidneys. A large percentage of pregnant women show some albumin in the urine, which is the result of a mild irritation from the pregnant condition, and may become so severe as to result in an acute nephritis with marked degeneration and necrosis of the renal epithelium.

Albuminuria of pregnancy comes usually in the latter half of pregnancy, and may be associated with red infarcts of the placenta.

Albuminuria, which resists treatment, and in the presence of other signs of toxaemia, should be treated by uterine evacuation.

Women with chronic nephritis are not more subject to eclampsia than others. Women with nephritic and cardiac disease are apt to abort early.

---

### The Significance of Albuminuria to the Ophthalmologist

Albert C. Snell, M. D.

The man with the ophthalmoscope has a particular advantage over his fellow practitioners, since, with the ophthalmoscope, he is able to look directly at the blood vessels and other structures in the fundus of the eye. In this way he is able to observe the exact changes which may have taken place in the retinal blood-vessel walls and in the surrounding tissue dependent on them for its nutrition.

Albuminuria means toxaemia, using the word toxaemia to mean that there is some irritating substance floating in the blood stream. The constant presence of these toxins are sure, sooner or later, to produce structural changes in the retinal blood-vessel walls, and in the retina itself. In fact all structures of the eye—the conjunctiva, the cornea, the sclera, the iris, cilary body, choroid, and optic nerve are also possible seats for lesions due to albuminuria, although the latter structures are not commonly affected by the toxins of albuminuria. Neither is the clinical picture of inflammation at all diagnostic in these parts. For this reason in the consideration of the significance of albuminuria to the ophthalmologist, I wish to direct your attention simply to the changes which may occur in the retina and its blood vessels.

The retinal lesions are either acute or chronic, depending on whether the albuminuria is acute or chronic. A good example of the acute form is the albuminuria of pregnancy. In this acute form one may either not see any changes in the fundus and the patients be more or less totally blind—a condition known as uremic amaurosis—or one may have an ophthalmoscopic picture that in itself is very characteristic, there being present an oedema about the optic nerve sheath, occasionally some small hemorrhages and enlarged veins, together with some loss of vision. In both of these acute forms the prognosis in regard to vision is usually very good. However, should the latter form continue over very many weeks the prognosis for vision becomes more grave. Should there be a recurrence of the albuminuric retinitis in a subsequent pregnancy, the vision may be completely and irrevocably lost. Here we often have a difficult problem to settle. Should we sacrifice the foetus to save the mother's sight or shall the mother take this great risk of blindness for the sake of a possible living child?

It is in the chronic form of albuminuria that one finds the interesting and significant changes in the retinal blood-vessels. For the sake of classification and simplicity I have been in the habit of dividing the chronic cases into three groups. In the first group are those in which the pathology is gross. Here we have the typical retinal findings of small or large areas of retinal degeneration, exudates, optic neuritis and arterial walls thickened, often appearing as "silver wire" arteries, the arterial walls appearing very white and thick. In the second group there is no gross pathology, although occasionally we may have present a few small hemorrhages. Arterial walls are slightly thickened, arteries themselves being very straight, and veins dilated and tortuous. In the third group, which is the very earliest stage of arterial changes, we note marked tortuosity of terminal arteries; and a decided depression of the vein at the position of the crossing of an artery over the vein. Occasionally we find the "locomotion" pulse. The latter group of cases have a very special value and significance in that they indicate a beginning arterio-sclerosis, and a stage that may be termed the pre-albuminuric. The ophthalmologist of course can not infallibly prognosticate an albuminuria from the fundus finding, but he can warn the family physician to be on the lookout for the possibility of such a condition. All this data studied with blood pressure, the urine, and the clinical findings will often help one to make a very early diagnosis at a time when the proper therapeutic and dietetic treatment is of most value.

I recently found the following review of an article in Knapp's Archives of Ophthalmology, March, 1914. "Out of twenty-eight cases of chronic nephritis studied by Gordinier (Exophthalmos a common symptom of chronic Bright's disease,) fourteen presented exophthalmos of varying degrees, associated with one or more of the accompanying ocular symptoms, such as those of von Graefe, Stellwag, or Moebius. He believes that the most probable explanation for these symptom is an irritation of the cervical sympathetic system by toxins floating in the blood stream as the result of renal insufficiency." (Archives of Diagnosis, April, 1912.) How many clinicians have noticed an exophthalmos in Bright's disease?

---

**Oxalic Acid in Foods.** Albaharry stated that oxalic acid is present in the tomato only in traces, the acid being the bimalate of potassium, which is also present in unripe grapes, cherries, raspberries, currants and sour apples. Malic acid is also present in oak leaves. Charles, Gaz. Hebd. des Sci. Med., July 20, 1913, confirms the above and states that oxalic acid is found in considerable quantities in sorrel, rhubarb stalks and some varities of peas.

---

**Relation of Thymus to Deaths After Thyroidectomy.** Klose, Berliner Klin. Woch., claims that such deaths are due to auto-intoxication from the thymus. Up to 1911, in Rohn's clinic, 8 deaths occurred in 130 cases of thyroidectomy for Basedow's disease. Since then, with conjoint removal of the thymus, no deaths have occurred in 200 cases.

